# Dissecting Loneliness in the Digital Age: An Insight into the Experiences of Medical Students Amid and Beyond the COVID-19 Pandemic

**DOI:** 10.12688/f1000research.141325.2

**Published:** 2024-06-26

**Authors:** Abdulqadir J. Nashwan, Rawan Alahmad, Ghazi Abu Afifeh, Nour Abu Afifeh

**Affiliations:** 1 Department of Public Health, College of Health Sciences, QU Health, Qatar University, Doha, Doha, Qatar; 2 Hamad Medical Corporation, Doha, Doha, 3050, Qatar; 3 Faculty of Medicine, Yarmouk University, Irbid, Irbid Governorate, Jordan; 4 Faculty of Medicine, The University of Jordan, Amman, Amman Governorate, Jordan

**Keywords:** Medical students, Loneliness, COVID-19, Digital age, Social media, Well-being

## Abstract

This narrative review explores loneliness among medical students, particularly heightened during the COVID-19 pandemic. This review aims to narratively describe how the digital age, both pre- and post-pandemic, influences loneliness and to assess the psychological effects of the pandemic on medical students. Our literature search, adhering to SANRA guidelines, scrutinized studies published in the last ten years focusing on loneliness among medical students. Our findings reveal that medical students experienced significant loneliness during the pandemic, attributed to virtual learning environments and decreased social interactions. Notably, the transition to online education has mitigated and exacerbated feelings of isolation. The review also highlights the dual role of social media in either alleviating or intensifying loneliness, depending on usage patterns and platform types. Overall, our study underscores the need for targeted interventions and support systems to address the mental well-being of medical students in the digital age and beyond, providing crucial insights for future research and policy-making in educational and psychological support frameworks.

## 1. Introduction

In December 2019, an outbreak of pneumonia with an unknown cause was reported in Wuhan, China. Investigation revealed that the cases were linked to the Huanan Seafood Wholesale Market. ^1^ Scientists were able to isolate a novel respiratory virus from respiratory samples, which was later identified as a new coronavirus belonging to the same family as the virus causing severe acute respiratory syndrome (SARS). ^2^ This new coronavirus was named SARS-CoV-2 and is responsible for the disease known as COVID-19. The rapid global spread of SARS-CoV-2 and the significant number of deaths led to the declaration of a pandemic by the World Health Organization in March 2020. ^2^ Beside the increased morbidity and mortality that resulted from the spread of this virus, the impact of the COVID-19 pandemic has been immense; as governments around the world have implemented lockdown measures to isolate the infection, resulting in economic consequences, and increased poverty worldwide. ^3^ In addition to that, extended periods of social isolation led to persistent feelings of loneliness and boredom, which can possibly have negative impacts on both physical and mental well-being on the long run. ^4^

The lives of numerous individuals have been profoundly affected by the emergence of COVID-19 and the subsequent measures done to control its transmission. Among these impacted groups are medical students. A study conducted in the US revealed that a substantial number of students had their clinical rotations cancelled or shortened by COVID-19. ^5^ Additionally, most students did not have in-person patient contact during the study period. Approximately, 75% of students acknowledged that their medical education had been significantly disrupted by the pandemic, and 84.1% of students agreed that COVID-19 pandemic affected their stress or anxiety levels. ^5^ Despite these challenges, students were able to find valuable learning opportunities through alternative methods such as telehealth, online courses, research, volunteering, and independent study. ^5^ But also, according to the study, 61.3 % of the respondents thought that medical students should continue with normal clinical rotations during this pandemic. In Saudi Arabia, a study found that 94.4% of medical students reported experiencing moderate to high perceived stress. Additionally, two-thirds of the students reported experiencing generalized anxiety symptoms, with 47% of them falling into the moderate to severe range. ^6^

Loneliness is defined by subjective feelings of social pain and isolation. It is considered a universal phenomenon in human experiences, and its emotional distress can have serious consequences. ^7^ Loneliness showed medium to large effects on all health outcomes according to a comprehensive review and meta-analysis conducted in 2020. ^8^ Those effects included physical and mental health outcomes, with the strongest impact being on mental health and well-being ( *i.e.,* depression, anxiety, suicidality, general mental health); it is especially crucial to prioritize the proper training of healthcare providers, enabling them to recognize and address their own feeling of loneliness. Given the fact that among medical residents, suicide ranks as the primary cause of death among males and the second leading cause among females. ^9^

As per a recent study examining loneliness among medical students, physician trainees, and faculty physicians, the findings indicate that loneliness is prevalent amongst people in the medical health industry, and that indeed includes medical students. ^10^

The COVID-19 crisis has resulted in the usage of digital platforms not only for entertainment purposes but also for educational and corporate reasons. ^11^ Several studies looked at the role of social media use during the COVID-19 pandemic. A Pakistani study had shown that while using social media during the COVID-19 crisis can be beneficial for emotional support, information gathering, and connecting with peers, it also takes a toll on mental health due to excessive usage. ^12^

Another study on Chinese college students revealed a connection between higher social media use and poorer mental health outcomes. Participants with high levels of disaster-related stress experienced greater depression when exposed to more disaster news on social media. Additionally, the relationship between social media use and mental health was mediated by negative affect, indicating that negative emotions played a role in this association. ^13^

In this review, we focus on the issue of loneliness as experienced by medical students, which is a significant concern, especially during the COVID-19 pandemic, considering the demanding nature of their studies and the impact of the pandemic on their education and well-being. The review also focuses on the digital age, by specifically examining loneliness in the digital age pre- and post- pandemic. It also gives insights into the psychological impact of the COVID-19 pandemic on medical students and how it intersects with loneliness. Overall, this narrative review holds importance by exploring an important topic, considering the digital age and the COVID-19 pandemic, and providing valuable insights for intervention, support, and future research.

## 2. Literature search

A systematic search strategy was employed that adhered to the SANRA guidelines. ^14^ PubMed, Scopus, PsycINFO, and Google Scholar, were searched using a combination of keywords and Boolean operators, such as “medical students,” “loneliness,” “mental well-being,” “virtual education,” “COVID-19 pandemic,” and related synonyms. The search was conducted in titles and abstracts, with inclusion criteria focusing on studies that specifically addressed aspects of loneliness, mental health, and virtual education among medical students, published in English, and peer-reviewed within the last ten years. Exclusion criteria ruled out studies not directly related to the topic. The screening process involved an initial review by title and abstract, followed by a full-text review.

## 3. The pre-COVID state of loneliness among medical students

Loneliness is a personal and psychological state that is linked to factors like social isolation, depression, introversion, or inadequate social skills. Studies have revealed that loneliness is most experienced among various social groups, including young adults (aged 18-29 years), older adults, individuals with physical or mental health conditions, those with low income, and people with different marital statuses, such as single, separated, widowed, or divorced individuals. ^15^

Medical students represent an important group deserving attention regarding the prevalence of loneliness and the factors influencing it. In a recent study focusing on loneliness among various medical professionals, including medical students, it was found that 20.9% of the participants of the medical students reported experiencing intense loneliness, indicated by scores of 2 or 3 on a scale ranging from 0 to 3. ^10^ In a study that looked at loneliness at universities, the average emotional loneliness score in medicine/health care study discipline was 0.847, while the mean social loneliness score was 0.302 (The general loneliness scale ranged from 0 to 6 (0–1 = not lonely, 2–4 = moderately lonely, 5–6 severely lonely). ^16^

A recent meta-analysis was conducted and revealed some risk factors of mental problems in medical students, and the results indicate that female medical students are at a higher risk for mental health issues, possibly due to pre-existing gender differences in mental distress. ^17^ Junior or preclinical students, who are at an early stage of medical education, are also at a higher risk for mental distress. Factors such as low social support, bad family relationships, economic troubles, pre-existing mental or physical illnesses, and COVID-19 infection or exposure are associated with mental problems among medical students. Unhealthy lifestyles, increasing substance use, irregular diet and sleep, and problematic smartphone and internet use are emerging risk factors that need further investigation. ^17^ Another study that involved Chinese medical students revealed that sophomores and junior students, neuroticism, high arousal symptoms, and the quality of support from friends were the risk factors for high loneliness profile. ^18^

Undoubtedly, social media stands as one of the most extensively utilized interactive technologies, so it is important to study how those social connecting platforms affect loneliness. ^19^ The primary purpose behind the development of social media was to establish connections between individuals in various parts of the world. ^20^ However, as technology brings people closer, the “alone together” phenomenon arises, where individuals feel isolated despite constant technological connections, and this can lead to serious problems such as depression, especially for people who have the -so called-, social network site (SNS) addiction. ^21^

SNS addiction is a phenomenon where individuals are so highly motivated to use social networking sites that it adversely affects their social activities, studies, work, relationships, and overall psychological health and well-being. ^22^ According to a meta-analysis that studied the SNS addiction problem in medical students, the prevalence of Internet addiction among medical students was five times higher than that of the general population. ^23^

According to a Chinese study, approximately 33.18% of the participating medical students were found to have an addiction to SNSs. Most of these students used social media platforms daily, with around half spending more than one hour per day on them. Among various demographic factors, the only influencing factor was the students’ grade level. In addition, the study demonstrated that SNS addiction has the potential to impact depression by affecting feelings of loneliness and unmet interpersonal needs. ^24^

Addressing loneliness among medical students is crucial to ensure their overall well-being and academic success. Loneliness can result in a sense of isolation and detachment, exerting adverse effects on a student’s mental health. This, in turn, may induce stress, potentially leading to detrimental impacts on academic performance. According to a study’s results, there was a positive association between loneliness and academic stress, meaning that as loneliness increased, so did academic stress. ^25^ On the other hand, there was a negative association between loneliness and psychological well-being, suggesting that higher levels of loneliness were linked to lower levels of psychological well-being. ^25^ In another study that exclusively evaluated the influence of family loneliness on medical professionalism measures ( *i.e.,* empathy, teamwork, and lifelong learning) among medical students, the findings revealed an inverse correlation between family loneliness and those measures; meaning that as family loneliness increased, the levels of empathy, teamwork, and learning measures tended to decrease among the medical students. ^26^

## 4. The impact of COVID-19 on loneliness among medical students

A study aimed to investigate the psychological challenges related to COVID-19 quarantine faced by medical students, with a focus on loneliness. ^18^ The researchers surveyed 1,478 participants using face-to-face online questionnaires, incorporating the University of California, Los Angeles (UCLA) Loneliness Scale and psychological characteristic scales. ^27^ The results revealed three distinct loneliness profiles: low loneliness (52.3%), interpersonal sensitivity loneliness (3.5%), and high loneliness (44.1%). Risk factors for high loneliness included being a sophomore or junior student, neuroticism, high arousal symptoms, and the quality of support from friends, while predictors of interpersonal sensitive loneliness were sophomore and junior students, openness, and conscientiousness personality traits. Conversely, good peer relationships and other support acted as protective factors for the low loneliness profile. However, limitations in the study, such as the single medical university’s participant selection, may affect the generalizability of the findings. In conclusion, targeted interventions addressing loneliness based on identified profiles and predictors in medical students are suggested, with timely support and strategies playing a crucial role in enhancing their mental wellbeing during challenging times like the COVID-19 pandemic.

Other researchers present a review of the advantages and disadvantages of virtual medical teaching for medical students during the COVID-19 pandemic, focusing on the shift to remote learning. ^28^ The study involved analyzing 201 articles, with 34 included, and conducting manual searches for additional references. Strengths of virtual teaching were identified, including improved access to diverse web-based resources, interactive teaching facilitating remote patient interactions, and open-access teaching with medical experts for staying updated on medical advancements. Peer mentoring also proved valuable. Conversely, weaknesses encompassed technical challenges, confidentiality issues, reduced student engagement, loss of assessments, and negative impacts on students’ mental well-being. Global inequalities in virtual teaching services further affected medical education. Participants strongly agreed that virtual learning increased their knowledge and stimulated learning, with most indicating a willingness to recommend and continue with this form of teaching. The advantages included the ease of accessing educational materials in preferred environments, accessibility to experts globally, and an opportunity to enhance virtual medical education. However, disadvantages included technical issues with audio and video, lack of clinical experience, loss of networking opportunities, cost and time burdens for faculties, potential boundary issues between work and home, and increased risks of isolation, anxiety, and boredom. As a researcher, this comprehensive analysis sheds light on the benefits and drawbacks of remote learning, underlining the significance of optimizing virtual medical education while addressing associated challenges for improved outcomes during unprecedented times.

Furthermore, a systematic review with meta-analysis aimed to investigate the potential increase in loneliness during the COVID-19 pandemic, considering the impact of measures like physical distancing. ^29^ The review identified 34 high-quality primary studies with 215,026 participants, including longitudinal and pseudo longitudinal designs. The meta-analysis revealed a small but significant increase in loneliness scores and prevalence rates compared to pre-pandemic times. The findings underscore the importance of addressing the issue of loneliness during the ongoing health crisis and highlight the need for further investigation into risk and protective factors to develop targeted interventions for mental and physical health support.

## 5. The role of social media during the COVID-19 era

A study investigated the impact of social media use during the COVID-19 pandemic, utilizing both quantitative and qualitative surveys with 307 participants. ^30^ The findings demonstrated that passive social media use was linked to increased loneliness and decreased life satisfaction. However, certain active social media engagement led to a rise in positive affect, while other active uses resulted in heightened feelings of loneliness. Platform-specific differences were also observed, with Twitter use being associated with increased feelings of loneliness. The qualitative results indicated a surge in social media use during the pandemic, providing opportunities for digital reconnection with old friends and family. These findings shed light on the diverse effects of social media use during COVID-19 and highlight the importance of understanding the nuances of online interactions on individuals’ emotional well-being.

In another investigation, a study explores the impact of COVID-19 on communication and collaboration in distance learning environments, along with the role of social media in this context. ^31^ The study collected data through an online survey from 234 students and tested research hypotheses. The findings indicate that increased use of Facebook for professional purposes enhances students’ communication and collaboration during distance learning. High activity on Facebook and LinkedIn is crucial for communication with educators. Active participation in distance classes and positive assessment of online tools also positively influence communication and collaboration among students, aligning with engagement theory. The research contributes to the distance learning literature by shedding light on the pandemic’s effects through the lens of engagement theory, and it offers practical implications for all participants in the educational process.

Furthermore, the COVID-19 pandemic and its containment measures have had a significant impact on mental health, especially during adolescence, a critical period for social and cognitive development. ^32^ Studies have shown that social deprivation during this time can lead to diverse mental health problems. ^33^ Lockdown measures during the early months of the pandemic threatened the mental health of youth due to distant learning, closure of leisure environments, decreased outdoor activities, and distress related to the pandemic. Several reviews have highlighted the immediate impact of the pandemic on young people’s mental health, showing increased anxiety, loneliness, stress, and depressive symptoms. ^34^ Adolescents responded to social distancing by spending more time online, particularly on social media platforms, to alleviate negative experiences. This increased screen time, along with digital technology use, has both positive and negative impacts on mental well-being. However, the specific link between digital media use and adolescents’ mental health during the COVID-19 pandemic has not been systematically studied. The present review aim to address this gap by focusing on the relationship between digital media use, mental health, and adolescents during the COVID-19 pandemic.

Moreover, another study examined the impact of social media use on mental health and well-being. ^35^ High frequency social media users, who used it several times daily, were found to have poorer mental health, overall quality of life, higher loneliness, and lower well-being compared to low-frequency users who used it daily or less frequently. Among those using social media daily or less frequently, 24.2% experienced emotional distress and poor overall quality of life. However, for high-frequency users, the proportions experiencing emotional distress and poor overall quality of life were significantly higher at 75.8% and 75.6%, respectively. The findings suggest that excessive use of social media may be associated with negative effects on mental health and overall well-being.

## 6. The post-COVID scenario and the evolving role of social media

In the post-COVID era, loneliness continues to be a prevalent and concerning issue among medical students. Approximately 44.1% of medical students fall into the high loneliness profile, indicating a significant proportion experiencing profound feelings of isolation and disconnection. Moreover, 3.5% of students are categorized under the interpersonal sensitivity loneliness profile, suggesting a subgroup struggling with interpersonal interactions. ^36^ This could be attributed to the pandemic’s impact on campus closures, limited social interactions, and increased academic pressure. Consequently, there is a pressing need for targeted support and interventions to address the mental health struggles of medical students in the post-pandemic era. By understanding and addressing loneliness, we can promote their overall well-being and ensure their success in both academic pursuits and future medical careers.

While numerous studies have explored the immediate impact of COVID-19 loneliness on the mental health of medical students, there is a lack of research on the long-term effects, this could be due to the temporal proximity of the lockdown termination. Regarding the short-term consequences, one study conducted in the United States found that medical students faced elevated levels of stress, burnout, and loneliness. ^36^ Students with preexisting mental health conditions are at a significantly higher risk, as they might encounter restricted availability of crucial treatments and services, leading them to adapt their care delivery methods, such as transitioning to virtual sessions. ^37^

These findings align with another study’s results, which indicated that students living alone during the pandemic were more prone to experiencing moderate to severe levels of anxiety and post-traumatic stress disorder (PTSD). ^38^

Additionally, the repercussions of pandemic-induced loneliness extended to sleep patterns. Strict social distancing measures and reduced interpersonal communication have intensified feelings of loneliness, exacerbating insomnia during the pandemic. ^39^

Due to quarantine measures, the role of social media has become more apparent. One finding suggests that social media may play a dual role in affecting loneliness during the pandemic. ^40^ For older adults, using social media may serve as a tool to prevent or reduce loneliness, particularly when faced with reduced social contact due to various factors related to aging. On the other hand, among younger individuals, excessive social media use may be associated with higher emotional loneliness, possibly due to social comparison tendencies and presenting a “liminal self” online. ^41^ Another study found that excessive and prolonged social media use, adopted as a coping strategy, resulted in negative consequences for mental health. ^42^

## 7. Mitigation strategies and interventions

**Figure 1.**
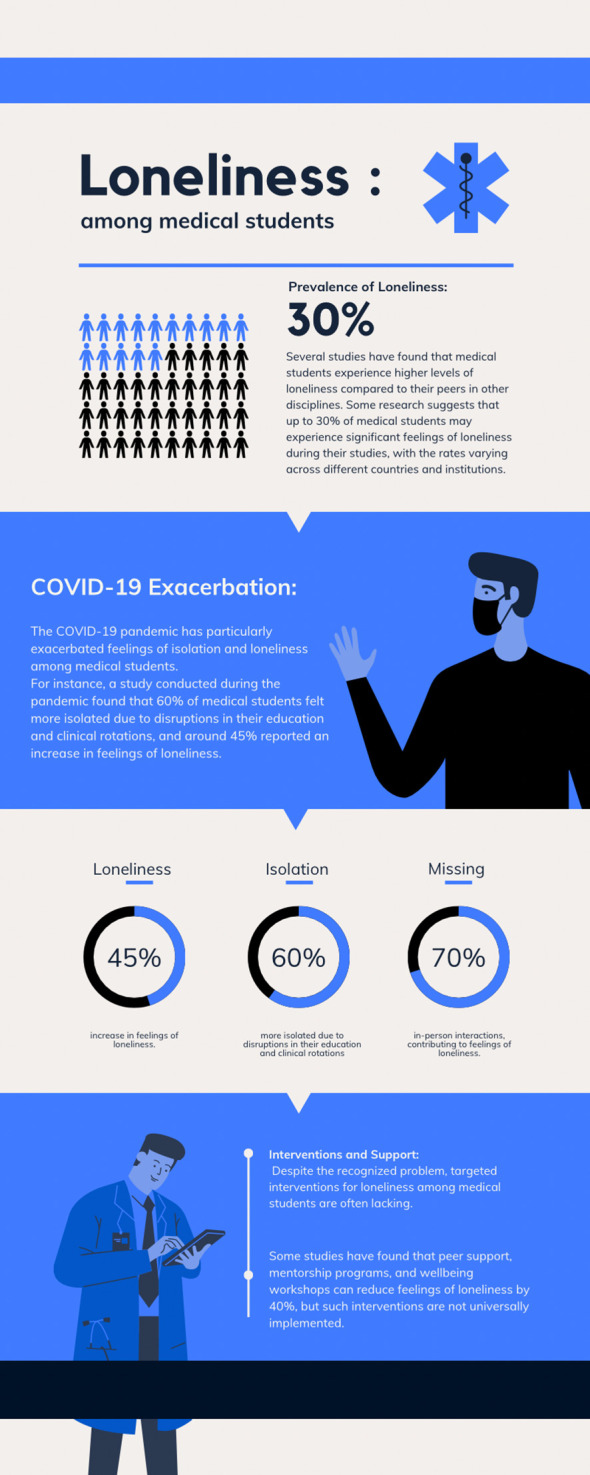
Facts and figures about loneliness among medical students. [This figure is an original figure produced by the authors for this review article.]

As medical students face increased social isolation, it becomes crucial to identify effective interventions to address loneliness and promote student wellness and resilience. ^43^ Various coping mechanisms have emerged, but focused interventions can significantly contribute to mitigating loneliness and enhancing students’ mental well-being. ^44^^–^^47^ Figure 1 illustrates facts and figures about loneliness among medical students.

Psychological interventions including cognitive-behavioral therapy (CBT), mindfulness-based interventions, social skills training, gratitude interventions, and reminiscence therapy have proven to be effective in reducing loneliness compared to control groups, with an overall effect size of 0.43; which would indicate that the psychological interventions (like CBT, mindfulness-based interventions, etc.) had a positive and moderate effect on reducing loneliness when compared to control groups. ^48^

Prolonged social isolation measures during the COVID-19 pandemic may have deprived individuals of the need to belong and connect with others, leading to increased feelings of loneliness. Correspondingly, having a robust network of peer support is essential in reducing the feeling of loneliness, as well as improving emotional well-being. ^49^^,^^50^

Medical schools play a crucial role in supporting student wellness. ^51^ They can incorporate evidence-based interventions into their programs to address loneliness effectively. ^52^ Group-based approaches, such as support groups, social interaction activities, and group-delivered psychoeducation, provide opportunities for students to connect, share experiences, and develop a sense of belonging. ^43^ Moreover, incorporating reflective exercises like mindfulness, meditation, or journal writing can be beneficial in reducing loneliness by promoting self-awareness and emotional well-being. ^43^

Furthermore, medical schools can harness the potential of online platforms, such as chat rooms or smartphone apps, to facilitate virtual connections and support, especially in situations where face-to-face interactions are limited. Social skills training can address maladaptive social cognition associated with loneliness by improving students’ ability to initiate and maintain social connections. ^43^

In addition to addressing loneliness directly, medical schools should focus on enhancing student resilience, which builds a protected personality that is able to tolerate challenging situations. programs may include workshops on stress management, coping strategies, and fostering a growth mindset. Encouraging a culture of open communication and emotional support among faculty and students can further contribute to enhancing student resilience. ^53^

## 8. Limitations

This narrative review has several limitations that should be considered when interpreting the findings. Firstly, while the review aims to synthesize a broad range of literature, including primarily English-language studies may have omitted relevant findings published in other languages, potentially introducing language bias. Secondly, the narrative approach, although beneficial for a comprehensive overview, does not allow for the quantitative synthesis of data which might lead to less precise conclusions compared to systematic reviews or meta-analyses. The reliance on published literature may also introduce publication bias, as studies with significant or positive results are more likely to be published than those with null or negative outcomes. Moreover, the definitions of key concepts such as “loneliness” and “social media use” were broadly interpreted and might vary between studies, potentially affecting the consistency of the results. Finally, the evolving nature of research on COVID-19 means that more recent studies or emerging data might not have been included, which could affect the review’s comprehensiveness and relevance over time.

## 9. Conclusions

Several studies have underscored the importance of addressing loneliness among medical students, particularly during the COVID-19 pandemic. The findings identified various loneliness profiles and risk factors among medical students, emphasizing the need for targeted interventions to improve mental well-being. Virtual medical teaching has been found to have both advantages and disadvantages, with some negative impacts on students’ mental health. More evidence further demonstrated an increase in loneliness during the pandemic, highlighting the necessity for mental and physical health support. Unique loneliness experiences of medical students compared to other students stress the need for specific understanding and support. The role of social media in loneliness is multifaceted. Passive use is linked to increased loneliness, while some active engagement can have positive effects. However, platform-specific differences exist, and excessive use, especially during adolescence, can have negative mental health effects. Understanding online interactions and promoting healthy social media usage is vital. Longitudinal studies to investigate the long-term impact of pandemic-induced loneliness on medical students’ mental health, career choices, and overall functioning are essential. There is an urgent need to develop specific interventions targeting medical students’ unique needs in the post-COVID era, as current interventions remain insufficient.

## Data Availability

No data are associated with this article.
