# Supplementary material for: Dissecting Loneliness in the Digital Age: An Insight into the Experiences of Medical Students Amid and Beyond the COVID-19 Pandemic
Source: F1000Res. 2024 Jun 26;12:1196. Originally published 2023 Sep 25. [Version 2] doi: 10.12688/f1000research.141325.2 (PMC11729188; doi:10.12688/f1000research.141325.2)
Supplement: Peer review history

# Reviewer report: Reviewer response for version 2

**DOI:** 10.5256/f1000research.167164.r353116

**Published:** 2025-01-13

**Authors:** Eugene Boon Yau Koh

**Affiliations:** 1 Department of Psychiatry, Faculty of Medicine and Health Sciences, Universiti Putra Malaysia, Serdang, Malaysia

Thank you for the invitation to review. 

 My review has a degree of bias, considering that the paper was initially written in 2023, reviewed in Q4 2024, and now myself. 

 Overall, the methodology was sufficient. It provided enough information for the narrative review to be generated. 

 The intervention slide provided contained imagery that might or might not be copyrighted. I agree with the previous reviewer that the authors should clearly state how the image was obtained. 

 The discussion is well elaborated for a narrative review.  However, this is where my bias will occur. Considering the pandemic was two years ago, is the review still relevant? Do we still need to look into these interventions, as the review stated, or should we explore the topic with a fresh lens? I am not suggesting that the authors rewrite this. It is what it is for a 2023 paper. I am writing this here for readers to consider critically when using the information.

Is the review written in accessible language?

Yes

Are all factual statements correct and adequately supported by citations?

Yes

Are the conclusions drawn appropriate in the context of the current research literature?

Partly

Is the topic of the review discussed comprehensively in the context of the current literature?

Yes

Reviewer Expertise:

Mental health intervention, psychiatry, psychotherapy, primary care mental health, lifestyle psychiatry.

I confirm that I have read this submission and believe that I have an appropriate level of expertise to confirm that it is of an acceptable scientific standard.

# Response

**Published:** 2025-01-13

**Authors:** Abdulqadir Nashwan

**Affiliations:** Hamad Medical Corporation, Qatar

Thank you for the thoughtful feedback on the manuscript.

 Regarding the review process, it is important to note that F1000Research follows an open and post-publication peer-review model, where the paper is published first and then undergoes review. This approach aligns with their commitment to transparency and rapid dissemination of research. Therefore, this should not be considered a weakness of the paper.

 For Figure 1, we have clearly stated in the manuscript that *"This figure is an original figure produced by the authors for this review article,"* addressing concerns about its originality.

 We appreciate the constructive points raised about the paper’s timeline and relevance. While the content reflects the situation as it was during the drafting in 2023, it offers a historical perspective for readers and researchers who may wish to build upon it or approach the topic with updated methodologies.

 Thank you once again for your review and valuable input.

# Reviewer report: Reviewer response for version 2

**DOI:** 10.5256/f1000research.167164.r327680

**Published:** 2024-10-15

**Authors:** Mahitab Abdelrahman

**Affiliations:** 1 Suez Canal University, Ismailia, Ismailia Governorate, Egypt

Overall, your research offers an insightful exploration of a concept that is seldom addressed within the healthcare field. However, there are a few minor concerns I would like to outline in more detail below:

 Literature Search: Please include specific examples of the exclusion criteria used to filter sources in your search.

 Introduction: The focus on Saudi Arabia in the introduction needs clarification. 

 Supplementary File: Consider attaching a table in a supplementary file listing the articles included in your study. The table should include the following details: Authors (Year), Article Title, Source Title, and Volume (Issue).

 Figures: Please provide the copyright details for the software or program used to generate the figure in your article.

 Limitations: As you acknowledged in your study's limitations, more recent articles were not included. Please specify the exact timeframe for the articles reviewed.

Is the review written in accessible language?

Yes

Are all factual statements correct and adequately supported by citations?

Yes

Are the conclusions drawn appropriate in the context of the current research literature?

Yes

Is the topic of the review discussed comprehensively in the context of the current literature?

Yes

Reviewer Expertise:

My areas of research are nursing administration, education, motivation, and leadership.

I confirm that I have read this submission and believe that I have an appropriate level of expertise to confirm that it is of an acceptable scientific standard, however I have significant reservations, as outlined above.

# Response

**Published:** 2024-10-16

**Authors:** Abdulqadir Nashwan

**Affiliations:** Hamad Medical Corporation, Qatar

- Overall, your research offers an insightful exploration of a concept that is seldom addressed within the healthcare field. However, there are a few minor concerns I would like to outline in more detail below:
  Literature Search: Please include specific examples of the exclusion criteria used to filter sources in your search.

Response: Thank you for your constructive feedback. I would like to clarify that this study is a narrative review, and no systematic search was conducted due to the heterogeneity of the literature available in this field. This approach allowed for greater flexibility in selecting relevant studies and perspectives across a broad range of publications, making it difficult to apply traditional exclusion criteria as seen in systematic reviews.

- Introduction: The focus on Saudi Arabia in the introduction needs clarification. 

Response: we gave only some examples from the available literature. 

- Supplementary File: Consider attaching a table in a supplementary file listing the articles included in your study. The table should include the following details: Authors (Year), Article Title, Source Title, and Volume (Issue).

Response: Again, this is a narrative review, and no systematic search was conducted. This table will not add much to the literature due to the huge variability of methods, settings, determinants, etc. 

- Figures: Please provide the copyright details for the software or program used to generate the figure in your article.

Response: We already mentioned this statement as per the journal's policy [This figure is an original figure produced by the authors for this review article.]

- Limitations: As you acknowledged in your study's limitations, more recent articles were not included. Please specify the exact timeframe for the articles reviewed.

Response: I agree with you, this is mostly due to the delayed peer review process by the journal.

# Reviewer report: Reviewer response for version 1

**DOI:** 10.5256/f1000research.154759.r229584

**Published:** 2023-12-20

**Authors:** Henok Dagne

**Affiliations:** 1 University of Tasmania (Ringgold ID: 3925), Hobart, Tasmania, Australia; 2 Department of Environmental and Occupational Health and Safety, Institute of Public Health, University of Gondar, Gondar, Ethiopia

Overall comments

 While the review raises important and relevant topics, evidence from qualitative studies is lacking. As experience is better captured using qualitative studies, including qualitative studies would have enriched the findings of this study. 

 There are concepts that need definition in the review. These include concepts such as pre-covid era and post-covid era, the digital age, engagement theory, healthy social media use, excessive social media use and others which are confusing unless appropriately defined.

 Detailed transparent search strategy: one limitation of this review is the lack of rigorous methodology. Although it is not expected to have every detail of the search strategy as a systematic review, the review could have benefited from a detailed method section.  Authors may include the search strategy and search terms used as an appendix in the revised version. As long as the search strategy is systematic, a clear description of how the articles have been screened, who screed them, and how disagreements have been solved has to be described. 

 The review did not include limitations. Including review limitations will strengthen the review so that readers will be able to read the findings keeping in mind the limitations. 

 Specific comments 

 Abstract: The abstract failed to summarise the review results. What is written in the abstract section is too general.

 Introduction: The introduction lacks focus. Authors may reduce significant portion of the following "In December 2019, an outbreak of pneumonia with an unknown cause was reported in Wuhan, China. Investigation revealed that the cases were linked to the Huanan Seafood Wholesale Market. Scientists were able to isolate a novel respiratory virus from respiratory samples, which was later identified as a new coronavirus belonging to the same family as the virus causing severe acute respiratory syndrome (SARS). ^2^ This new coronavirus was named SARS-CoV-2 and is responsible for the disease known as COVID-19. The rapid global spread of SARS-CoV-2 and the significant number of deaths led to the declaration of a pandemic by the World Health Organization in March 2020. ^2^ Beside the increased morbidity and mortality that resulted from the spread of this virus, the impact of the COVID-19 pandemic has been immense; as governments around the world have implemented lockdown measures to isolate the infection, resulting in economic consequences, and increased poverty worldwide. " and focus more on the impact of the pandemic on loneliness.

 Lots of information is cited from a single study reference #5. This may not always be wrong, but authors could have included other studies, too. 

 The definition provided in paragraph 3 is misplaced. Authors may define the concept before the last statement of the first paragraph. 

 The introduction should show the relevance of the review by uncovering the reasons that led to undertaking the narrative review. 

 Along with this, why authors have opted for narrative reviews over the other types of reviews could have been justified. 

 Literature search: SANARA guideline is not a guide lane for search strategy it is a reporting quality appraisal tool. " systematic search strategy was employed that adhered to the SANRA guidelines." is confusing.  Authors may clearly show how many articles were screed for full text and how many of them were included in the last narrative review. 

 3. The pre-covid state of loneliness

 The first statement is repetition.

  The next statement "Studies have revealed that loneliness...." cited a single reference 15. 

 6.  The post-COVID scenario and the evolving role of social media

 "While numerous studies have explored the immediate impact of COVID-19 loneliness on the mental health..." cite at least some of these studies.

 Figure 1: How did the authors reach at numbers displayed in the figure as 30%, 45%, 60%, 70% etc. ?

  8. conclusion

 "Unique loneliness experiences of medical students compared to other students stress the need for specific understanding and support." This needs to be supported with evidence in the main text. While the authors mentioned medical students are more prone to loneliness as compared to the general population, the case is not well developed for comparing medical students with students of other disciplines.  

 "Longitudinal studies to investigate the long-term impact of pandemic-induced loneliness on medical students' mental health, career choices, and overall functioning are essential. this statement has a number of issues; first, is it really unique for medical students? second, whether studies on the long-term effect of loneliness on career choice are available from the main text. It appears in the conclusion section out of nowhere. 

 Also "There is an urgent need to develop specific interventions targeting medical students' unique needs in the post-COVID era, as current interventions remain insufficient."  The literature in the main text did not show current interventions remain insufficient.

Is the review written in accessible language?

Yes

Are all factual statements correct and adequately supported by citations?

Yes

Are the conclusions drawn appropriate in the context of the current research literature?

No

Is the topic of the review discussed comprehensively in the context of the current literature?

Partly

Reviewer Expertise:

Public Health, Epidemiology, Reviews (Scoping, methodological, systematic and meta-analysis)

I confirm that I have read this submission and believe that I have an appropriate level of expertise to confirm that it is of an acceptable scientific standard, however I have significant reservations, as outlined above.

# Response

**Published:** 2024-05-25

**Authors:** Abdulqadir Nashwan

**Affiliations:** Hamad Medical Corporation, Qatar

Response to Overall Comments:

 - We acknowledge the importance of qualitative evidence in capturing experiential aspects. However, this is not a systematic review and is not meant to capture all the published literature.

 - We agree that clarity is essential for understanding. However, this will be beyond the aim and focus of this review. 

 - We appreciate the feedback on our search strategy and methodology. Again, this is a narrative review and not expected to follow a systematic search strategy same as systematic reviews.

 We will add a section on our review's limitations. "This narrative review has several limitations that should be considered when interpreting the findings. Firstly, while the review aims to synthesize a broad range of literature, including primarily English-language studies may have omitted relevant findings published in other languages, potentially introducing language bias. Secondly, the narrative approach, although beneficial for a comprehensive overview, does not allow for the quantitative synthesis of data which might lead to less precise conclusions compared to systematic reviews or meta-analyses. The reliance on published literature may also introduce publication bias, as studies with significant or positive results are more likely to be published than those with null or negative outcomes. Moreover, the definitions of key concepts such as "loneliness" and "social media use" were broadly interpreted and might vary between studies, potentially affecting the consistency of the results. Finally, the evolving nature of research on COVID-19 means that more recent studies or emerging data might not have been included, which could affect the review’s comprehensiveness and relevance over time."

 Abstract: We will revise the abstract to ensure it concisely summarizes the key results of the review, focusing more on specific findings rather than general statements. "This narrative review explores loneliness among medical students, particularly heightened during the COVID-19 pandemic. This review aims to narratively describe how the digital age, both pre- and post-pandemic, influences loneliness and to assess the psychological effects of the pandemic on medical students. Our literature search, adhering to SANRA guidelines, scrutinized studies published in the last ten years focusing on loneliness among medical students. Our findings reveal that medical students experienced significant loneliness during the pandemic, attributed to virtual learning environments and decreased social interactions. Notably, the transition to online education has mitigated and exacerbated feelings of isolation. The review also highlights the dual role of social media in either alleviating or intensifying loneliness, depending on usage patterns and platform types. Overall, our study underscores the need for targeted interventions and support systems to address the mental well-being of medical students in the digital age and beyond, providing crucial insights for future research and policy-making in educational and psychological support frameworks."

 - The introduction will be sharpened to focus specifically on the pandemic's impact on loneliness, reducing the background details on COVID-19’s origins and global impact, which, while informative, detract from the main topic.

 - We will broaden our citation base to include multiple sources, ensuring a more robust representation of existing research and reducing the reliance on single study references.

 - We will rearrange the placement of key definitions to appear earlier in the text, ensuring they are established before extensive discussion.

 -  The rationale for opting for a narrative review over other review types will be clearly articulated, highlighting its relevance and effectiveness in addressing the research questions.

 - We will correct the confusion regarding the SANRA guidelines and clarify the actual process used in the literature search, including the number of articles screened and included.

 - Figure 1 Clarification: we mainly relayed on several estimates such as https://www.sciencedirect.com/science/article/pii/S0165032722012319#t0005 and https://www.ingentaconnect.com/content/ben/cpsr/2015/00000011/00000002/art00003.

 - We will ensure that statements in the conclusion about the unique experiences of medical students are well-supported with evidence from the main text. We will also clarify the scope and necessity of proposed longitudinal studies and interventions tailored to medical students, ensuring the discussion is directly tied to evidence presented in the review.
